# Current State-of-the-Art and Unresolved Problems in Using Human Induced Pluripotent Stem Cell-Derived Dopamine Neurons for Parkinson’s Disease Drug Development

**DOI:** 10.3390/ijms22073381

**Published:** 2021-03-25

**Authors:** S. A. Antonov, E. V. Novosadova

**Affiliations:** Institute of Molecular Genetics, National Research Center “Kurchatov Institute”, Kurchatov Square 2, 123182 Moscow, Russia; novek-img@mail.ru

**Keywords:** induced pluripotent stem cells, dopamine neurons, differentiation, drug screening, parkinson’s disease

## Abstract

Human induced pluripotent stem (iPS) cells have the potential to give rise to a new era in Parkinson’s disease (PD) research. As a unique source of midbrain dopaminergic (DA) neurons, iPS cells provide unparalleled capabilities for investigating the pathogenesis of PD, the development of novel anti-parkinsonian drugs, and personalized therapy design. Significant progress in developmental biology of midbrain DA neurons laid the foundation for their efficient derivation from iPS cells. The introduction of 3D culture methods to mimic the brain microenvironment further expanded the vast opportunities of iPS cell-based research of the neurodegenerative diseases. However, while the benefits for basic and applied studies provided by iPS cells receive widespread coverage in the current literature, the drawbacks of this model in its current state, and in particular, the aspects of differentiation protocols requiring further refinement are commonly overlooked. This review summarizes the recent data on general and subtype-specific features of midbrain DA neurons and their development. Here, we review the current protocols for derivation of DA neurons from human iPS cells and outline their general weak spots. The associated gaps in the contemporary knowledge are considered and the possible directions for future research that may assist in improving the differentiation conditions and increase the efficiency of using iPS cell-derived neurons for PD drug development are discussed.

## 1. Defining the Midbrain Dopaminergic Neurons

The midbrain dopaminergic (DA) neurons are of particular interest for modern molecular biology due to their selective death in Parkinson’s disease (PD), a widespread incurable neurodegenerative disease. The progressive loss of these cells in PD produces dopamine deficit in their basal forebrain targets causing severe motor impairments and leading to complete disability at the late stage of disease [1]. To date, the causes of neuronal degeneration in PD and methods for its prevention are unknown. Therefore, only symptomatic treatment options aimed to compensate the decreased dopamine levels in the striatum are currently available [2]. The search for mechanisms underlying the emergence and progression of PD and the need to devise the efficient treatments have motivated profound studies on the development and functioning of midbrain DA neurons that made these cells one of the most well-studied neuron type in mammalian brain.

DA neurons are distinguished by the expression of the set of genes involved in the synthesis (*TH*, *AADC*), transport (*DAT*, *VMAT2*), and degradation (*MAO*, *COMT*) of dopamine. According to conventional classification [3] that relies on anatomical distribution of DA cells, these neurons are represented by eight distinct groups in the mammalian brain (A8–A16) and a single (A17) group in the retina [4]. Additionally, a dispersed population of telencephalic DA neurons is present in the primate brain, which was not included in this classification [4]. Three of these groups—A8 (retrorubral field), A9 (compact part of the substantia nigra, SNc), and A10 (ventral tegmental area, VTA) are located in the midbrain. The closely positioned ventral midbrain A9 and A10 groups sharply differ in their functions and afferent connections [4]. Functionally, the A9 group is associated with locomotion, while A10 group regulates the motivation and emotional state. A remarkable difference between these two cell groups is the higher vulnerability of A9 neurons in PD and their higher vulnerability to DA toxin 1-methyl-4-phenyl-1,2,3,6-tetrahydropyridine (MPTP), the commonly employed agent for inducing DA neuron death in animal studies. On the other hand, A10 neurons were found to be much more resistant under these conditions [5,6]. Notably, it is the loss of A9 neurons that produces the primary motor symptom complex of PD that includes tremor, rigidity, and bradykinesia [6]. Consequently, the generation of pure or enriched cultures of A9 DA neurons from pluripotent stem cells (PSC) is of a special interest for PD studies in vitro.

Recent studies have revealed multiple differences between A9 and A10 neurons at the molecular level. Thus, *SOX6* transcription factor was shown to be selectively expressed by A9 DA neurons [7], while the expression of *Otx2* and *Nolz1* is restricted to A10 group in the adult midbrain [7,8]. These genes were shown to distinguish A9 and A10 progenitors already at the early stages of brain development, raising the possibility for their selective generation from pluripotent stem cells (PSC) in vitro [7]. However, according to La Manno et al., the midbrain DA neuron subtypes emerge from a common progenitor pool, and their segregation takes place only during the maturation stage, as a result of interaction of these cells with their specific environment. In light of this theory, the directed differentiation of PSC into the individual DA progenitor subtypes with consequent generation of pure of A9/A10 neuron cultures are not feasible [9]. Despite these controversies, the obtainment of pure or enriched cultures of DA neuron subtypes may be also potentially achieved by their separation from mixed populations (e.g., by FACS) or by forced expression of subtype-specific genes.

The gene expression analyses of single DA neurons in adult ventral midbrain have revealed the heterogeneity in A8–A10 groups, and distinguished five neuron subtypes within these populations [10]. Besides the previously described differential Otx2 expression [8], DA subtypes also show different patterns of *Aldh1a1*, *Adcyap1*, *Calb1*, and *Ndnf* expression [10]. Importantly, the distribution of these DA subtypes does not completely overlap with the established anatomically discrete zones. For instance, according to Poulin et al., the most vulnerable to MPTP toxicity 1A subtype primarily resides in the ventral part of SNc. However, the scattered cells with this molecular fingerprint are also present within the A8 area [10]. Interestingly, the genes exerting differential expression between midbrain DA neuron subtypes are associated with cell survival. Specifically, aldehyde dehydrogenase (*Aldh1a1*) is involved the disposal of potentially toxic dopamine metabolites (i.e., DOPAL), calbindin is a Ca^2+^-buffer protein that potentially contributes to the maintenance calcium homeostasis [11], and neuron-derived neurotrophic factor (Ndnf) was shown to promote the survival of endothelial cells [12]. These DA subtype-specific proteins have not been considered yet as markers for the characterization of PSC-derived DA neurons.

To date, the *GIRK2* (also known as *KCNJ6*, *Kir3.2*) gene is the most widely used marker for the discrimination of midbrain DA neuron subtypes in vitro. A successful generation of SNc DA neurons from PSC is often claimed on the basis of positive immunostaining for GIRK2 in tyrosine hydroxylase (TH) expressing cells [13,14,15]. However, multiple studies have demonstrated that *GIRK2* is neither specific for A9 neurons nor for the midbrain DA neurons in general [5]. Thus, it was established that *GIRK2* is also expressed by telencephalic DA neurons [16], while only quantitative differences in the expression level of this gene exist between A9 and A10 subtypes [17].

According to several studies, the mesencephalic A9–A10 neurons possess a dual transmitter character, being capable of secreting glutamate in addition to dopamine [18]. The observations supporting this hypothesis include fast excitatory postsynaptic potentials generated in DA neuron targets (that cannot be explained by DA transmission through the metabotropic dopamine receptors) and immunoreactivity of DA neurons to glutamatergic markers phosphate-induced glutaminase and glutamate itself [19,20]. Furthermore, the majority of midbrain DA neurons were shown to express type 2 vesicular glutamate transporter (*Vglut2*) during their development and after injuries in the adult brain [21], or even at normal conditions, according to others [22]. Interestingly, *Vglut2* expression in these cells was shown to be associated with their vulnerability to DA toxins [21]. To our knowledge, the dual transmitter properties were never investigated in DA neurons derived from PSC in vitro.

The synthesis and degradation of dopamine are known to be associated with constitutively high level of oxidative stress. Dopamine degradation by monoamine oxidase, cyclooxygenase, and tyrosinase is associated with generation of reactive oxygen species, and the non-enzymatic dopamine oxidation produces toxic quinone compounds [23]. Thus, to sustain their functioning during the whole human lifespan, DA neurons possess the remarkable resistance to the oxidative stress.

Both A9 and A10 DA neurons display unique pacemaker activity consisting of autonomous action potential (AP) firing [24,25]. Importantly, the mechanisms for AP generation are different between these two DA neuron subtypes and rely upon l-type calcium channels in A9 but not in A10 neurons [5]. The high energy demands for maintaining this activity in A9 cells impose additional burden upon electron-transfer chain and may contribute to increased vulnerability of these cells mitochondrial stress [26].

## 2. The Critical Steps of Midbrain DA Neuron Development

We only briefly outline here the primary differentiation cues involved in the development of midbrain DA neurons that constitute a basis of PSC differentiation protocols, while the detailed reviews of developmental biology of DA neurons are provided elsewhere [11,27,28].

The appearance of the first cells of neural lineage in the developing embryo is initiated by neural induction. These neuroepithelial cells derive from a group of competent ectodermal cells as a result of simultaneous inhibition of BMP and TGFβ signaling pathways by Noggin, Chordin, and Cerberus secreted proteins [29,30]. The nascent neuroepithelial cells of the embryo and their PSC-derived homologs (neural progenitor cells, NPC) possess “default” anterior character that commit them to differentiate into the forebrain derivatives, and must be subjected to caudalizing signals such as FGF-family proteins and retinoic acid to adopt fates of other CNS regions [29,31].

The emergence of soluble morphogen gradients in the nervous system leads to its regionalization along vertical and horizontal planes. In the rostro-caudal axis, the neural tube forms series of neuromeres—discrete transverse segments with different gene expression patterns and differentiation potentials. Dorso-ventral regionalization of neural tube demarcates dorsal, alar, basal and floor plates that differ in the repertoire of neuron subtypes they are able to produce [32]. The development of midbrain DA neurons takes place in the floor plate of the midbrain neuromere [33]. Pivotal roles in the specification of these cells are played by FGF, Hedgehog [27] and Wnt signaling pathways [34]. The spatial Shh gradient in the neural tube regulates neuroepithelial cell ventralization, that attain basal or floor plate phenotype depending on the Shh level [35]. The notochord-derived Shh induces secondary Shh expression domain in Foxa2^+^ floor plate through a positive feedback loop [36]. Importantly, the Shh-expressing ventral midbrain domain was shown to serve origin not only for A9/A10 DA neurons, but also the cells of red nucleus, oculomotor accessory nucleus, and several other non-DA neuron subtypes [37]. This large potential of ventral midbrain progenitors raises a reasonable concern on maximum purity of DA neuron cultures that can be achieved in practice via directed differentiation of PSC-derived floor plate cells. Relatively high proportions of DA neurons that are commonly achieved in the modern differentiation protocols [14], assume that differentiation of midbrain floor plate NPC into non-DA neuron types may rely on additional growth factors besides those provided in the differentiation medium.

The midbrain position within neural tube is established by soluble morphogen FGF8, an indispensable factor for the differentiation of DA neurons in this region [27]. Besides the midbrain, the *FGF8* and *Shh* expression domains also overlap in the basal forebrain and interbrain during the embryonic development. In light of this it appears surprising that a combined FGF8/Shh treatment selectively promotes DA fate in PSC-derived multipotent neural progenitors [38,39,40].

An important role in the specification of midbrain DA neurons in vivo is played by the Wnt signaling pathway [41]. Wnt1 and Wnt5a ligands are expressed in the midbrain floor plate and Wnt1 is required for specification and survival of a subpopulation of DA neurons in this region [27]. According to early studies in mouse ES cells, Wnt factors are not only dispensable for generation of DA neurons but may even inhibit this process [42]. However, it was later demonstrated that supplementing the differentiation medium with Wnt proteins, in fact, increases proportion of DA neurons generated from mouse ES cells [43]. Moreover, further studies have shown that activation of Wnt signaling during the differentiation of human PSC is essential for derivation of authentic ventral midbrain progenitors [14,16].

The interplay between Hedgehog-, FGF- and Wnt-signaling establishes the defining gene expression pattern of midbrain floor plate progenitors that includes *Foxa2*, *Lmx1a/1b*, *En1/2*, *Nurr1*, and *Pitx3* [11,28]. The specification of DA progenitor subtypes was shown to be regulated by retinoic acid and possibly FGF20 (Fibroblast growth factor 20) that promote the differentiation of A9 progenitors, while in their absence these cells attain the A10 fate [11].

The differentiative divisions of midbrain floor plate NPC give rise to post-mitotic DA neurons, which migrate radially from the proliferative ventricular zone, initiate neurite branching and ultimately establish their afferent connections with striatal and cortical targets [44]. In vitro, the generation of post-mitotic neurons is achieved by transferring floor plate NPC into mitogen-free terminal differentiation medium [14,15] The survival of newborn DA neurons is dependent on neurotrophic factors BDNF and GDNF [44]. Concentration gradients of these factors in the developing nervous system ensure the elimination of neurons showing aberrant migration trajectories or failing to establish the proper target connections. Importantly, only GDNF is essential for survival of these cells in adult brain [27]. The in vitro cultures of iPS cell-derived DA neurons are routinely fed with large excess of these neurotrophic factors during their differentiation, maturation and further maintenance [14,45]. In this context, the development of synthetic small-molecule BDNF and GDNF mimetics would significantly reduce the costs for derivation of DA neurons (and other neuron subtypes) from human PSC.

**Figure 1:**
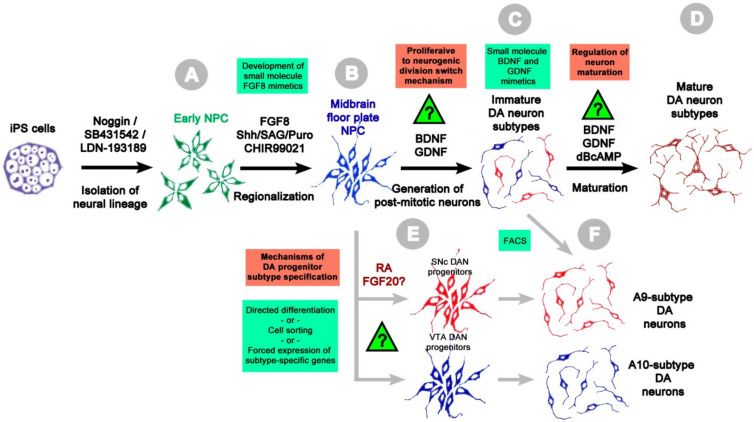
The general differentiation sequence of human iPS cells into the midbrain DA neurons. The crucial stages of in vitro differentiation protocols are shown by black arrows with small molecules and growth factors for inducing the neural lineage progression listed above. Grey arrows show potential routes for generation of pure or enriched cultures of specific midbrain DA neuron subtypes. Question signs indicate processes proceeding in vitro in uncontrolled/autonomous manner through unknown mechanisms highlighted in the red boxes; understanding of these mechanisms is critical for overcoming heterogeneity and asynchronicity of neuronal differentiation of iPS cells. The suggestions for optimizing or refining the differentiation conditions are shown in the green boxes. Withdrawal of iPS cells from self-renewing conditions and their treatment with TGFβ antagonists either in monolayer culture or in suspension EBs induces the differentiation of early multipotent NPC (**A**). After isolation and expansion, these cells are regionalized into the midbrain floor plate NPC (**B**) through activation of Hedgehog, Wnt and FGF8 signaling. While several commercially available small molecules can efficiently substitute recombinant Shh and Wnt1/Wnt5a, no FGF8 mimetics have been developed to date. Alternatively, midbrain floor plate NPC can be directly obtained from iPS cells by the overexpression of *Lmx1a*, *Foxa2* and *Nurr1*, and other midbrain-specific TF genes. (**C**) Generation and (**D**) functional maturation of post-mitotic DA neurons are performed by their continuous maintenance in presence of neurotrophic factors BDNF and GDNF, and optionally dibutyril cAMP (dBcAMP) and Notch antagonists (e.g., DAPT) to promote their survival and differentiation. However, due to the lack of control over these processes the resulting cell populations are heterogenous, consisting of neural progenitors and different DA neuron subtypes at various stages of maturation. The development of small molecule mimetics of FGF8, BDNF and GDNF proteins would be beneficial for reducing the costs of generation of iPS cell-derived neurons. The generation of floor plate progenitor subtypes (**E**) can be potentially carried out by employing selective growth factor-based conditions, forced expression of subtype-specific TFs or isolation from mixed cultures by FACS. These progenitor subtypes could be further differentiated into corresponding subtypes of post-mitotic DA neurons (**F**). Alternatively, pure or enriched cultures of A9 and other midbrain DA neuron subtypes could be obtained by their isolation from mixed DA neuron cultures using FACS at the terminal differentiation stage. The detailed description of each stage is provided in the text. BDNF—brain derived neurotrophic factor, GDNF—glial cell derived neurotrophic factor, FGF8—fibroblast growth factor 8, FGF20—fibroblast growth factor 20; Puro—puromorphamine, RA—retinoic acid, Shh—sonic hedgehog.

In sum, as evidenced by in vitro studies, the listed sequence of lineage progression events (Figure 1) is both essential and sufficient for realizing complete differentiation program of midbrain DA neurons. In fact, all other aspects of differentiation of these cells are allowed to proceed in vitro in uncontrolled autonomous mode. Notably, the regulation of several critical processes in this differentiation sequence including the switch from proliferative to differentiative divisions of neural progenitors, the mode of segregation of DA neuron subtypes and, as it will be discussed below, the regulation of DA neuron maturation remains largely unexplored (Figure 1). Lack of control over these differentiation stages significantly contributes to heterogeneity and asynchronicity of generation of mature human midbrain DA neurons. Deciphering of mechanisms underlying these processes would be highly beneficial for improving the efficiency of PSC-based models in PD research.

## 3. Directed Differentiation of ES and iPS Cells into the Midbrain DA Neurons

Two types of PSC are currently available: embryonic stem (ES) cells, which, as their name implies, are derived from human embryos, and induced pluripotent stem (iPS) cells generated by reprogramming of different somatic cells (e.g., fibroblasts, lymphocytes). The use of latter cell type has several obvious advantages as their derivation is possible from any adult donor and does not raise ethical concerns. ES cells were instrumental for pioneering studies of differentiation programs, and their well-characterized phenotype is now used as a gold-standard for establishment of new iPS cell lines [46].

The methods of directed differentiation of PSCs into DA neurons are continuously refined, but the significant time consumption required for the generation of post-mitotic neurons remains a persistent drawback of most current protocols [47].

In general, two main approaches are currently employed for directed differentiation of PSC into specific neuron subtype that rely on mimicking the differentiation conditions in vivo by using soluble morphogens and growth factors [14,15,48,49] or using the forced expression of lineage-specific transcription factors (TFs). Most commonly, the protocols relying on the latter approach are complemented by the use of growth factors [13,50,51,52,53].

### 3.1. Differentiation Protocols Based on the Use of Soluble Morphogens

Basically, the differentiation of PSC into specific types of neurons can be subdivided into three sequential stages: (1) the neural induction giving rise to the multipotent NPC, (2) the regional specification of committed NPC (the midbrain floor plate cells committed to differentiate into subtypes of neural cells specific to the corresponding brain region), and (3) the terminal differentiation and maturation of post-mitotic neurons [54]. The described sequence of events during the generation of midbrain DA neurons from human PSC and the unresolved issues at each stage of current in vitro differentiation protocols are shown on the Figure 1.

Significant progress in the field of directed differentiation of PSCs was achieved by introducing the small molecule growth factor mimetics—the selective agonists or inhibitors of specific intracellular signaling pathways [55].

A neural induction in PSC cultures can be achieved through the formation of embryoid bodies (EBs), the suspension cellular aggregates consisting of the derivatives of three germ layers. The application of recombinant FGF2 to EBs was shown to promote the expansion of NPC [45]. A more efficient and widely used approach is based on simultaneous application of BMP/TGF signaling inhibitors (the so-called dual SMAD inhibition) [56] that produces rosettes of multipotent NPC in monolayer culture, or their clusters in EBs [57]. These NPC can be readily expanded after their dissection or may be directly subjected to further differentiation into neurons. Compared to other methods, this approach affords NPC with higher neurogenic potential [58]. Most commonly a small molecule inhibitor SB431542 is used as a TGF antagonist [56,58], while BMP signaling is suppressed by recombinant Noggin [56], or small molecules Dorsomorphin [58,59] and LDN-193189 [15,51,57] (Figure 1).

Although the replacement of recombinant proteins with small molecules can significantly reduce the cost of performing directed differentiation, their efficiency can significantly differ from endogenous substances. For instance, Nolbrant et al. advised against replacing recombinant BMP-scavenger protein Noggin with BMP-signaling antagonist LDN-193189 due to formation of non-neural cell types in the resulting cultures [49]. On the other hand, the small-molecule Wnt agonist CHIR99021 was shown to be superior to recombinant Wnt1 and Wnt3a proteins for derivation of floor plate cells from human ESC by providing stronger activation of corresponding signaling pathway [14]. Another small molecule SAG (Smo AGonist) successfully substitutes recombinant Shh for generation of DA neurons from human and other primate PSCs [59,60]. While both compounds show similar ventralizing potency [59], the strongest effect on NPC ventralization is achieved when SAG is combined with a low Shh concentration [61].

The generation of committed midbrain progenitors from multipotent early NPC indispensably involves the use of FGF8 and Shh (or Shh mimetics SAG or puromorphamine) [14,62,63] (Figure 1), unless the target cell types are induced by forced expression of region-specific genes (see below). Both Shh and FGF8 exert pleiotropic effects, and their expression domains overlap in several areas outside ventral midbrain. These morphogens form spatiotemporal gradients in the developing neural tube, where the quantitative differences of their levels establish the different NPC lineages [37]. Notably, the FGF8 concentration of 100 ng/mL [15,16] that is commonly utilized in protocols for the directed differentiation of PSC into DA neurons appears arbitrary as its correlation with endogenous FGF8 level in the developing ventral midbrain is questionable.

Given the positive feedback that induces Shh expression in floor plate cells in response to the notochord-derived Shh signal [37], the endogenous Shh secretion by NPC cultures in response to its exogenous application would contribute to heterogeneity of differentiating cultures. That way, the descendants of correctly ventralized floor plate NPC may attain different degree of ventralization after withdrawal of exogenous Shh supplementing. Controversial data exists on Shh effects on NPC proliferation in different models are: for instance it was reported that Shh acts as a mitogen for mouse NPC [64], while its high concentrations were shown to inhibit the proliferation of chick floor plate progenitors [65]. The necessity of Hedgehog signaling inhibition for NPC transition from self-renewal to generation of post-mitotic DA neurons in vivo [34], indicates that withdrawing the exogenous supply of Shh may be insufficient for inducing synchronous and uniform differentiation of floor plate cells into the post-mitotic neurons in the in vitro cultures.

Similar to Shh, FGF8 does not selectively promote the midbrain fate in naïve neuroepithelial cells. For instance, along with mesencephalic derivatives, FGF8 was shown to induce the differentiation of rhombencephalic cells from caudal forebrain tissue explants [66]. Accordingly, FGF8 also promotes specification of telencephalic neuron subtypes and the brainstem 5-HT neurons from PSCs [67,68] and is involved in the differentiation of caudal spinal cord progenitors. [69].

The timing of Shh/FGF8 treatment is of critical importance in determining their effects in early NPC: thus, it was shown that delayed application of these factors in NPC cultures obtained from human ES cells apparently induces differentiation of telencephalic DA neurons, as the resulting TH^+^ cells lack expression of midbrain-specific gene *Engrailed1* (*En1*) and retain primitive bipolar morphology [70]. Commonly, TH^+^ neurons generated from PSC-derived NPC upon Shh/FGF8 treatment are considered mesencephalic, even though it was previously established that TH may show artifactual expression in the primary forebrain neuron cultures as a consequence of high neurotransmitter plasticity of these cells under the in vitro conditions [71]. Thus, only the co-expression analyses may reliably confirm the generic midbrain phenotype.

According to Kriks et al. the immediate specification of midbrain floor plate cells from PSC-derived early NPC is crucial for generation of DA neurons with authentic mesencephalic molecular fingerprint [14]. These authors have demonstrated that induction of NPC and their subsequent regionalization by FGF8/Shh must be carried out in parallel with the Wnt-signaling pathway activation for obtaining cells expressing the full repertoire of the midbrain floor plate genes, including *Foxa2* and *Lmx1a*. This strategy not only ensures acquiring the correct midbrain phenotype by NPC and their progeny, but also significantly increases the proportion of generated DA neurons by suppressing the differentiation of other neuron subtypes [14].

However, it was later reported this floor plate NPC-based differentiation protocol still does not provide the sufficient specificity for obtaining the ventral midbrain DA neurons, as FOXA2^+^/LMX1A^+^ progenitors also generate the diencephalic subthalamic nucleus neurons. In this regard differentiation selectivity was improved by optimizing the timing of FGF8 treatment [49].

The single-cell transcriptome analysis of DA neuron cultures obtained from ESCs and iPSCs by method of Kriks et al. [14] has revealed the presence of three of mesencephalic DA neuron subtypes described in vivo [9]. However, at the same time point (63 days of differentiation) several subtypes of NPC were also identified in these cultures, indicating the asynchrony of generation of post-mitotic neurons. These results underscore the need for a further refinement of the terminal differentiation conditions, as the growth factor withdrawal is apparently not sufficient for suppressing NPC self-renewal and promoting their differentiative divisions [9].

The enrichment of the DA neuron populations can be achieved by fluorescence-activated cell sorting (FACS). However, applying this approach is complicated by the paucity of available surface markers specific for ventral mesencephalic NPC and DA neurons, although several marker subsets for enrichment of these cell types were reported. Thus, Suzuki et al. have shown that CD184^high^/CD44^−^ NPC isolated by FACS produce significantly higher yield of DA neurons compared to initial unsorted NPC cultures. [63]. Also, Schöndorf et al. employed the sorting of CD24^high^/CD29^−^ /CD184^−^ /CD44^−^ /CD15^−^ cell fraction to enrich the cultures of post-mitotic DA neurons. Importantly, it was demonstrated that this procedure does not affect the neuronal viability [72].

Despite significant advances in the development of directed differentiation protocols, their reproducibility may vary in a substantial range [47,51]. For instance, DA neuron yield was reported to amount from 8 to 80 percent under the same differentiation conditions [73,74,75,76,77,78]. This paradox can be explained by two possible reasons. Firstly, the activity of recombinant proteins and even small molecules may differ depending on the manufacturer and production batch. To obtain more consistent results, it is advisable to titrate every new batch of employed differentiation compounds, as it was recommended, for example, for Wnt-agonist CHIR99021 [49]. The second possible reason is the unequal sensitivity to morphogens of different PSC lines due to the different expression levels of corresponding signaling pathway components. This phenomenon has been clearly demonstrated in different human iPS cell lines [51,79,80]. Accordingly, it was reported that Shh and CHIR99021 concentrations should be titrated individually for each particular iPS cell line [49]. Such optimizations of the differentiation protocol are especially important in the experiments involving comparison of neurons derived from different iPS cell lines. Neglecting these line-specific features poses a risk of misinterpreting the experimental results by considering the differences in DA neuron numbers caused by suboptimal differentiation conditions as the manifestation of pathological phenotype. At the same time, considering the critical importance of concentration of morphogens in the nutrient medium and their application timing, any modifications of the existing protocols could produce qualitative changes in the composition of the obtained cell populations. Thus, line-specific optimization of differentiation conditions should be accompanied by comprehensive morphological, immunocytochemical and functional evaluation of generated cell cultures.

### 3.2. Differentiation Protocols Based on Genetic Manipulations

Two transgene-based options are available to promote the generation of midbrain neurons from PSC. These approaches rely on the introduction of genetical constructs bearing either the midbrain-specific TF genes or their promoters driving the expression of reporter proteins. The use of these reporter constructs opens the opportunity to isolate the desired cell types from heterogeneous populations. In the addition to providing the efficient routes for generation of midbrain NPC, the latter approach significantly contributed to our understanding of developmental aspects of these cells. The forced expression of midbrain-specific TFs allows one to bypass the internal limitations of the PSC differentiation potential and the stochasticity of their behavior, ensuring homogeneous differentiation into the target neuron type. However, the constitutive overexpression of any given proneural gene is artificial and may potentially lead to phenotypic deviations of generated cells from their in vivo counterparts. In this regard, the inducible constructs that can be shut down after achieving the desired differentiation stage are preferable. As the integrating vectors impose risk of the off-target effects in the cell’s genome, the delivery of genes of interest by adeno-associated viruses, mRNAs or other non-integrating methods represent safer options.

The regionalization of NPC by forced expression of the floor plate genes is apparently the most widely used method for promoting the generation of DA neuron progenitors. This approach allows overcoming the variability of differentiation efficiency between iPS cell lines for obtaining consistent DA neuron yields.

*Lmx1a* overexpression was utilized in several studies to induce the differentiation of PSC into committed ventral midbrain NPC [51,81]. Notably, using Lmx1a-GFP and Pitx3-GFP human iPSC reporter lines, it was found that Lmx1a^+^ NPC possess lower proliferative potential and a higher propensity to differentiate into DA neurons, yielding more homogeneous cultures of DA neurons in vitro [82]. However, it is known that, in contrast to *Pitx3*, which is specific for mesencephalic DA neurons [37,83], *Lmx1a/b* genes are also expressed in diencephalic DA neuron progenitors (A11–A15) [60] and *Lmx1a* expression is also characteristic for glutamatergic neurons of adjacent to the midbrain subthalamic nucleus [84]. Furthermore, Nefzger et al. FGF8/Shh-induced differentiation of Lmx1a^+^ NPC derived from human ESC primarily generates basal telencephalic GABAergic neurons [85], and therefore is not recommended for use as a specific midbrain DA progenitor marker. Accordingly, the transduction of iPS cells with non-integrating adeno-associated viral vectors carrying single ventral midbrain-specific genes *Lmx1a*, *Nurr1*, or *Pitx3* afford similar yields of DA neurons, which make up 30–40% of the total neuron number [51].

The derivation of committed mesencephalic DA progenitors was also achieved by lentiviral and retroviral transduction of *Nurr1* and *Foxa2* genes [50]. This dual gene-based approach contributes to specificity of differentiation process, as it was previously shown that sorting of human PSCs-derived NPC expressing the floor plate marker *Foxa2* alone does not promote the differentiation of DA neurons [86].

The direct differentiation of human iPS cells into DA neurons was performed by transfection with *Atoh1* (*atonal homolog 1*) and *Ngn2* (*neurogenin 2*) mRNAs. mRNA transfection was performed in several rounds to achieve the required expression level of target genes and was accompanied by Shh/FGF8 treatment. The authors reported obtaining cultures that consisted of 90% TH^+^ DA neurons co-expressing GIRK2 and DAT, as well as the midbrain markers Lmx1a and Foxa2 [53].

In sum, the forced expression of pro-DA differentiation genes offers attractive prospects for accelerated specification of DA neurons and reducing the expenses for their obtaining. The strategies with the simultaneous delivery of multiple genes provide higher specificity and homogeneity of the resulting populations.

The presence of undifferentiated progenitors and non-DA neuron subtypes in DA neuron cultures may negatively impact the experimental performance and the obtained data quality. The contaminating cell types introduce stochastic component to transcriptome and single-cell PCR analyses and complicate the electrophysiological measurements. The use of reporter gene that is selectively expressed in post-mitotic DA neurons is a convenient solution to detect these cells in heterogenous cultures. Thus, reporter iPS cell lines expressing *GFP* [87,88] or the brighter reporter protein *mOrange* [89] under the control of *TH* promoter were developed using the CRISPR Cas9 genome editing system. These genetic modifications facilitated the identification of DA neurons in mixed neuron cultures and xenotransplants, and even enabled their isolation in a viable state by FACS [89]. These reporter lines offer a simple and efficient approach for selective targeting of DA neurons for patch clamp, fluorescence imaging, laser microdissection, and other single cell analyses.

In sum, the described methods for directed differentiation protocols for iPS cells unravel two alternative routes for derivation of midbrain DA neurons, each with its pros and cons. The protocols relying on overexpression of midbrain-specific genes offer more robust and less time-consuming output. However, in all cases, the resulting cultures are assumed to contain multiple DA neuron subtypes and only the casual analysis of the A9 subtype-specific features of DA neurons (GIRK2 expression) was performed. The differential vulnerability of midbrain DA neuron subtypes in PD underscores the urgent need for development of methods for selective generation of A9 neurons from PSC, as a more relevant model in context of PD research. The subtype-specific properties of DA neurons also outline the need for careful assessment of experimental data obtained in the studies employing mixed DA neuron cultures. However, the separate derivation of A9/A10 midbrain DA neuron subtypes from iPS cells was not reported to date and theoretical basis for such procedures is currently lacking.

## 4. In Vitro Maturation of Human DA Neurons

The acquisition of functional maturity by post-mitotic neurons relies on the complex interactions of intracellular signaling pathways [90]. The maturation of neurons was shown to be promoted by stimulation of their electrical activity [91,92], activation of neurotransmitter receptors [93], co-culturing of these cells with astrocytes and neurons from rodent species [94,95] and interaction of neurons with extracellular matrix [96,97]. Despite the extensive amount of empirical data, the general mechanisms regulating the timing of neuronal maturation are obscure [98].

Multiple authors reported that maturation of human neurons in vitro requires an extended culture time [90,99], ranging from 50 to 70 days [14,15,100] for midbrain DA neurons. However, even at day 50 of maturation the transcriptome analysis has demonstrated the persistence of proliferating NPC and immature neurons in PSC-derived cultures [101].

The present PSC differentiation protocols consider the neuronal maturation as an autonomous process that primarily depends on culture duration [14,100]. The maturation of PSC-derived DA neurons was shown to be accelerated by increasing intracellular cAMP levels [102], and a respective widespread practice exists to employ its cell-permeant analog dibutyryl-cAMP at the terminal differentiation stage of these cells [14]. There is also a common practice of supplying the terminal differentiation medium with cAMP itself [45,103], despite its inability to pass through the cytoplasmic membrane and absence of data on the efficiency of this treatment. The Notch pathway antagonists DAPT and LY411575 were shown to promote the neuronal differentiation and maturation [104,105], and thus are utilized in some PSC differentiation protocols [98].

However, there is a lack of systematic studies on intracellular signaling pathways involved in human neuron maturation. The existence of internal clock mechanism determining the maturation time of neurons was suggested [98], but its molecular basis remains elusive.

The use of *synapsin-I* promoter-driven GFP reporter was suggested for the rapid assessment of maturation of iPS cell-derived DA neurons, and the selective expression of this reporter was demonstrated in DA neurons possessing spontaneous activity [51]. However, in our own studies, we observed the immature ionotropic neurotransmitter receptor properties in TH^+^ iPS cell-derived DA neurons showing robust spontaneous activity, indicating the poor correlation between these phenotypic traits [106]. In general, multiple investigators have emphasized that expression of established neuronal markers significantly precedes the appearance of mature functional properties, such as action potential firing and synapse formation in human PSC-derived neurons [90,107]. Despite these reports, the expression of these proteins (e.g., NeuN/Fox-3 and MAP2) is still frequently considered as a hallmark of neuronal maturity [15].

The transcriptome analysis of single PSCs-derived GABAergic and glutamatergic neurons performed after the patch-clamp measurements provided new insights into the maturation-associated changes of gene expression patterns, revealing 350 transcripts upregulated in mature neurons [108]. These genes await their evaluation as the potential maturity markers.

To date, electrophysiology remains the primary and most reliable method for assessing the functional maturation of human neurons [108]. The resting potential of −70–90 mV and the ability to fire bursts of evoked action potentials (APs) are general mature neuron properties that are most widely employed for estimating the functional state of PSC-derived neurons [98,107]. However, the innate low performance of electrophysiology experiments and the need for specialized equipment limit the use of this method in PSC research. To date, the in-depth electrophysiological studies were carried out mainly on PSC-derived cortical neurons [90,98,99], while much less data is available for midbrain DA neurons [109]. Therefore, an urgent need exists to develop alternative methods for assessing the functional maturity of human neurons and to establish its correlation with gene expression profile.

Ca^2+^-imaging was utilized in multiple studies for studying the human neuron maturation [110,111]. This method was successfully employed for monitoring general neuron excitability, the pacemaker activity of the DA neurons [100] as well as functioning of neurotransmitter receptors [106], and may complement or even substitute the patch clamp measurements [112] for studying the maturation of these cells.

The maturation of neurons is the most time-consuming part of protocols for the derivation of neurons from human PSC. Yet even the long-term differentiation conditions do not allow to obtain the uniform mature human neuron cultures [101].

The stimulation of neuronal maturation in vitro by different exogenous factors highlights its non-autonomous character that is not hardwired to in vivo developmental timing.

Elucidating the triggers of maturation of neurons to decipher the gene network associated with elusive “inner clock” mechanism and to find the routes to override it may not only provide a basis for developing more rapid in vitro differentiation protocols but would also improve our global understanding of construction and repair of neuronal circuits. The correlative studies of gene expression patterns, electrophysiological measurements and calcium imaging involving both human PSC-derived and primary rodent neuron cultures are required to clarify these mechanisms.

## 5. 3D Cultures

Three-dimensional culture methods constitute a rapidly advancing field of modern stem cell biology. Under the 3D culture conditions, the NPC aggregates exhibit striking capacity to produce multicellular structures that consist of different neural cell types and attain hallmarks of brain tissue-specific organization as a consequence of ordered cellular proliferation and differentiation governed by intrinsic mechanisms. The complex microenvironment within the brain organoids promotes the development of neuroglial contacts and advanced neural networks that are not observed in 2D culture conditions. Thus, 3D cultures afford the opportunity of studying the pathogenesis of neurodegenerative diseases in the conditions mimicking human brain structure [113] and obtaining the models of human diseases that could not be recreated using conventional two-dimensional cultures and transgenic animal models [111]. It was reported that brain organoid cultures could be maintained in vitro for more than one year [114], thus offering unique capabilities for studying neurodegenerative diseases with the late onset and continuous asymptomatic course [115].

For the establishment of brain organoid cultures, the PSC-derived EBs or neurospheres are propagated on semi-permeable membranes at the air–liquid interface [116,117], synthetic scaffold surfaces or decellularized extracellular matices [118,119], or are embedded in the gel medium and maintained in suspension culture [62,120,121,122], of which the latter method is the most widely employed.

Most commonly, the organoids are cultured in the polymerized Matrigel^TM^ medium [62], that primarily consists of laminin, collagen and nidogen/entactin proteins [123]. Notably, among these proteins, only laminin constitutes a part of embryonic brain extracellular matrix (ECM), while in the adult brain these proteins are expressed exclusively in blood vessel basement membranes, but are absent in perineuronal nets and interstitial matrix [124] consisting of proteoglycans, tenascin, and hyaluronic acid [125]. Since ECM is known to play an important role in regulation of the neural cell migration and neurite outgrowth [126], and is considered to be involved in neurodegenerative processes [121], a further refinement of organoid culture methods may be required to better recapitulate the in vivo conditions.

Heterogeneous synthetic scaffolds consisting of multiple components are being developed and investigated for their usefulness for 3D culture of brain cells. For instance, it was reported that composite silk fibroin-hydrogel matrices promote the segregation of gray and white matter zones in 3D cultures [127]. Also, the promising capabilities for long-term organoid cultures were demonstrated for decellularized brain-derived ECM. Thus, it was demonstrated that fetal porcine brain decellularized ECM support the growth of neuronal cultures for two years without losing its structural integrity [119].

One of the major problems of organoid cultures consists in the absence of circulatory system, with consequential limited oxygen and nutrient supply to the central region of the organoid and causing the development of inner necrotic zone [128]. It was reported that a significant reduction of this necrotic area in midbrain organoids can be achieved by using flow culture system [120]. For achieving higher similarity of organoid structure to the native brain tissue, a co-culturing method was developed for neural and endothelial cells derived from iPS cells of the same donor. The formation of extensive capillary network inside the organoid stroma was reported in these conditions, providing significant improvement of cell survival upon transplantation of these organoids into the rodent brain [129]. Though it was demonstrated that 3D cultures, in particular in the air-liquid interface culture system, can be maintained in stationary conditions [122], generation of larger organoids with more pronounced tissue-specific structure and better cell survival is achieved by using the roller flasks [111], orbital shakers [62,130], or flow systems [120,121]. Apparently, the use of such equipment demands for extra space in CO_2_-incubators, and implies the need for additional investments for cell culture facilities.

The fluorescence imaging of cells in 3D cultures is technically more complex compared to 2D cultures. The light scattering by tissue and off-focus fluorescence in brain organoids that achieve significant sizes (>1 mm) [62,122] limit the usefulness of conventional wide-field fluorescence microscopy to imaging of cells in the vicinity of organoid surface, while confocal or multiphoton microscopy are required for studying of cells in the depth of such specimen [131,132]. Besides, the slow diffusion of fluorescent probes in the dense tissue may cause gradient staining artifact in large organoids (the staining intensity being proportional to cell’s distance from surface facing the dye solution, causing overstaining of outer cells during the time required for dye to reach the organoid core), limiting the possibilities of imaging and quantitative comparisons between the cells located at different depths of the specimen (e.g., in the Ca^2+^- or Cl^−^-imaging). Hence, the simple bath-loading of fluorescent indicator dyes may be impractical for larger organoids, and more sophisticated techniques such as microinjection may be required for labeling of their inner cells, as it is performed in the in vivo imaging [133]. The imaging of suspension organoid cultures also requires the specially designed chambers ensuring the specimen immobility during the application of investigated compounds and other experimental manipulations. Due to the same concerns the morphological and immunofluorescence assaying of organoids require the use of histology [134] or confocal microscopy [135,136]. The whole mount immunofluorescent staining of organoids [135] or preparation of their histological sections [134] are significantly more time-consuming and laborious compared to staining of 2D cultures. Considering these features, it can be assumed that 3D models more likely would complement the conventional 2D culture studies rather than replace them in the in vitro studies.

## 6. Artificial Ageing of Neurons

The higher incidence of PD in the elderly evidences for existence of a link between cellular ageing and the pathogenesis of PD [137,138]. For instance, in accordance with this hypothesis the dysfunction of the PD-associated nuclear protein SATB1 produces the senescent phenotype in DA neurons [138]. Combined with intense oxidative metabolism [23,137] and a significant load of calcium-transporting systems [26], the ageing of DA neurons aggravates their vulnerability to stressing insults.

Thus, the use of senescent neurons for modelling PD in vitro may reproduce the conditions for the development and progression of this disease in the human brain more accurately than their convenient cultures. Due to “erasing” of the donor’s age in iPS cells [139,140], it was suggested that neurons directly induced from the somatic cells might be a more appropriate model than iPS cell-derived neurons for studying of age-related human diseases [139]. However, it must be noted that age-associated changes in donor’s non-neural somatic cells (that are inherited by induced neurons) may not correspond to those in the brain.

To date, two options have been described for inducing the aged phenotype in iPS-cell derived neurons. The first one is based on overexpression of progerin, a mutant form of lamin A protein associated with premature ageing in humans. The accumulation of progerin in the nuclear membrane disrupts chromatin organization and nuclear membrane functions, leading to manifestation of a senescent phenotype in the cell (reviewed in [141]). It was shown that transfection of DA neurons with progerin-encoding mRNA induces the characteristic ageing-associated changes in these cells. For instance, progerin-expressing DA neurons display genomic DNA damage, increased levels of reactive oxygen species in mitochondria, neurite degeneration, and the accumulation of neuromelanin. Progerin-transfected neurons show substantial similarity to neurons in the aged rat brain, highlighting the specificity and efficiency of this method [140]. Its usefulness for neurodegenerative disease modeling was further corroborated by another study employing progerin transfection into GABAergic neurons derived from iPS cells of Huntington’s disease patients. Strikingly, these cells not only attained the general features of senescence but also developed the phenotypic hallmarks of the corresponding disease [142]. However, the primary progerin-induced lesions including the disturbed chromatin architecture and nuclear membrane functions that are not observed in PD may interfere with the evaluation of targeted therapy drugs. In sum, progerin overexpression in DA neurons holds a significant potential as a model for studying the pathogenesis of PD but may be a less suitable option for screening of antiparkinsonian drugs.

Alternatively, the artificial ageing of cell cultures could be achieved by hydroxyurea (HU) treatment [143,144]. The application of this ribonucleotide reductase inhibitor disrupts the synthesis of deoxyribonucleotides thus suppressing the DNA repair in non-dividing cells. HU treatment of human mesencephalic DA neurons derived from iPS cells of patients with sporadic PD was shown to induce an array of phenotypic traits associated with neuodegeneration. The 4-day HU application significantly increased the expression of stress-associated genes, inhibited neurite outgrowth and induced activation of pro-apoptotic enzymes in these cells [145]. Methodologically, the use of HU is a simpler approach in comparison with forced progerin expression since the former does not depend on transfection efficiency and is less time consuming.

## 7. The Use of iPS Cell-Derived Neurons in Drug Development and Screening

**Figure 2:**
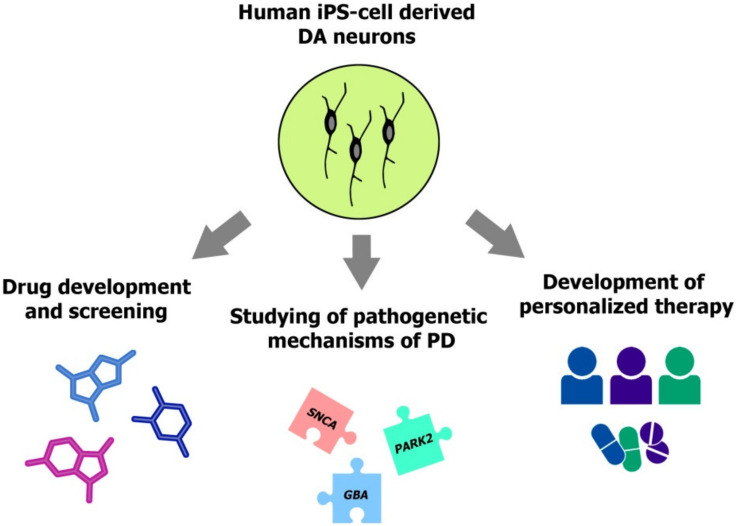
In vitro studies based on human iPS cell-derived DA neurons. Drug development and disease modeling with these cells represent rapidly advancing fields of modern research. The personalized therapy is a perspective area for future studies relying on patient-specific neurons, but due to paucity of currently available treatment options for PD and absence of approved targeted drugs it was not implemented into the practice to date.

Due to unavailability of primary cultures of human DA neurons and their progenitors, iPS cells represent the unique source of these cell types for the in vitro studies. The generation of iPS cells from patients with various forms of PD has provided the basis for new generation of drug screening assays, development of personalized therapy in PD and studying pathogenetic mechanisms of this disease (Figure 2). To date, a wide array of iPS cell lines were generated from donors with hereditary and sporadic forms of PD (Table 1), opening the unprecedented opportunities for studying the roles of specific genes and their interplay in the development and progression of this disease. The manifestation of PD-associated phenotypic traits including aberrant alpha-Synuclein accumulation, formation of Lewi bodies, impaired mitophagy and mitochondrial functions in DA neurons derived from PD patients (Reviewed in [146,147]) make these cells a highly promising models in PD research.

Due to the absence of karyotypic abnormalities in iPS cell-derived neurons, these cells possess fundamentally higher similarity to in vivo neurons in their responsiveness to exogenous substances compared to transformed cell lines, thus providing physiologically more relevant data in toxicological assays. Moreover, the PSC-derived DA neurons do not suffer from batch-to-batch variability in contrast to primary neuron cultures. Ultimately, compared to brain-derived neural stem cells the PSC-derived NPC can be extensively passaged without losing their neurogenic potential and their sensitivity to regionalizing cues [180]. Thus, iPS cell-based drug screening has several marked advantages over the other existing in vitro models.

The possibility of using iPS cells for drug screening attracts significant attention in the modern literature with multiple reviews devoted to this issue [181,182], and the general aspects of studying pathogenesis of PD and the development of iPS cell-based novel approaches for its treatment are reviewed in [183]. Particularly, we previously developed the basic test system employing human iPS-derived NPC and neurons for primary assessment of neuroprotective substances [184]. Recently, a 3D-culture based high throughput screening system was described that offers improved data reproducibility through standardization of organoid size [185].

In recent years, the use of DA neurons derived from iPS cells was instrumental for development of a number of promising antiparkinsonian drugs. Thus, a small molecule glucocerebrosidase modulator S-181 was shown to increase the activity of this enzyme in DA neurons of healthy donors and restore its impaired function in neurons of PD patients. S-181 efficiency was confirmed in vivo, as its administration to mice was shown to decrease the brain alpha-synculein level [186]. Human DA neurons generated from iPS cells were shown to be a valuable model for studying the role of oxidative stress in the development of PD and assessment of potential antioxidants [187]. For instance, neuroprotective effects of a small molecule antioxidant NC001-8 were demonstrated using iPS cell-derived DA neurons that were challenged with hydrogen peroxide [188].

Sherman et al. performed a large-scale screening of compounds affecting neurite outgrowth in human iPS cell-derived neurons elegantly employing immunofluorescent staining and segmentation-based image analysis. [80] The identified hits in this study may find their future use in the restoration of damaged neural circuits in PD and other neurodegenerative diseases.

The intact intracellular signaling pathways and the native expression profile of receptors and secondary messenger systems in iPS cell-derived neurons ensure that effects of the investigated compounds in these cells could be reliably translated to in vivo activity. For instance, Spathis et al. employed this model to evaluate the potential of targeting the nuclear receptor Nurr1 with a novel Nurr1: RXRα-complex agonist BRF110 for PD treatment [189]. *Nurr1* is essential for development and survival of mature DA neurons, while its impaired expression is known to induce their death and is associated with PD [190]. Nurr1 is considered an orphan receptor because its endogenous agonists are unknown [191]. Using DA neurons generated from iPS cells of PD patients, Spathis et al. showed that BRF110 increases the expression level of genes associated with dopamine synthesis and promotes cell survival under the oxidative stress conditions. Importantly, it was established that BRF110 produces similar effects in mice, highlighting the reliability and high predictive value of data obtained from iPS cell-based models [189].

The realization of the potential of human iPS cell-derived neurons for screening and drug development is hindered, however, by extensive duration and complexity of current differentiation protocols. The labor-intensive steps such as the manual isolation of iPS cell colonies for derivation of EBs and dissection of neural rosettes negatively impact the performance rate of these studies. Also, the significant expenses of growth medium containing multiple recombinant proteins in the course of neuronal maturation are reflected in high costs of the assays involving iPS-cell derived neurons. In this context, the development of synthetic small-molecule compounds that may substitute growth and neurotrophic factor proteins is highly desirable. In addition, the repeated medium exchange procedures during the longtime terminal differentiation stage are associated with potential risks of microbial contamination, complicating the large-scale utilization of this model. Thus, optimization of protocols for generation of DA neurons from iPS cells to achieve faster and more robust differentiation outcome, in particular by employing the overexpression of DA progenitor-specific genes can help increase the efficiency of iPS cell-based studies.

## 8. Concluding Remarks

In sum, the advent of iPS cells has provided unprecedented capabilities for studying the pathogenesis of PD in vitro and screening the antiparkinsonian drugs. Significant advances have been achieved to date in the development of methods for the generation of ventral mesencephalic DA neurons from PSC. Yet, the asynchrony of generation of post-mitotic neurons leading to the heterogeneity of the resulting cultures remains a weak spot of the current directed differentiation protocols. This problem is evidenced by variable DA neuron yields reported in different studies using the same differentiation conditions. In this regard, a better understanding of mechanism promoting the switch from proliferative to differentiative divisions of NPCis required. Furthermore, the titration of factors and small molecules used in the differentiation protocols and optimization of differentiation conditions for individual iPS cell lines may be necessary for obtaining consistent yields of neuron type of interest and improving the robustness and efficiency of drug screening assays.

The unequal vulnerability of midbrain DA neuron subtypes in PD emphasizes the need for development of protocols for selective generation of A9 neurons for disease modeling. The separation of DA neuron subtypes from mixed populations by cell sorting methods is currently not feasible due to the absence of subtype-specific surface markers, making the detailed analysis of the midbrain DA neuron surface molecules highly anticipated. While the in vivo data indicates the existence of distinct subtype-specific DA neuron progenitor pools in the midbrain floor plate and provides clues of their differentiation mechanisms, their derivation in vitro has not been reported to date. Whether these progenitor pools are composed of genuinely different cell types, or their differences arise from their spatial positioning in vivo, is a matter of further research, in particular involving in vitro assessment of their differentiation potential. In addition, the methods for characterization of PSC-derived midbrain NPC and DA neuron subtypes require further improvement by implementing the data of recent developmental studies in rodent models.

The rich capabilities for functional assays offered by human iPS cell-derived DA neuron cultures can be hardly overestimated. However, their use is confounded by the extended maturation time, an issue inherent to all current differentiation protocols. Our understanding of mechanisms regulating neuronal maturation remains rudimentary and the currently employed maturation conditions for human neurons solely rely on the empirical data. Thus, systematic analyses of external factors that affect the rate of human neuron maturation are essential to provide the rationale for the development of more rapid and efficient differentiation protocols.

## Figures and Tables

**Table 1:** The list of currently available iPS cell lines derived from donors with different forms of hereditary PD.

Gene	Mutation	Reference
*SYNJ1*	R258Q	Vanhauwaert [148]
*SNCA*	Gene triplication	Devine [149], Byers [150], Flierl [151]
	A53T	Chung [152], Ryan [153]
*LRRK2*	G2019S	Tolosa [154], Nguyen [155], Reinhardt [156], Skibinski [157], Sanchez-Danes [158], Sandor [159], Hsieh [160], Novosadova [161], Cooper [162]
	R1441C	Cooper [162], Hsieh [160]
	R1441G	Hsieh [160]
	Y1699C	
*PARK2*	V324A	Chung [163], Miller [140]
	Exon 2 deletion	Shuvalova [164]
	Exon 3 deletion/R42P	Zhong [165]
	Exon 3 and/or 4 deletion	Shaltouki [166]
	Exon 3 and/or 5 deletion	Jiang [167]
	Homozygous exon 3 deletion	Zhong [165], Ren [168]
	Compound heterozygous deletion of exones 3 and 5	Zhong [165], Ren [168]
	Exon 2–4 and/or 6–7 deletion	Imaizumi [169], Suzuki [63]
*PINK1*	Q456X	Chung [152], Seibler [170], Morais [171], Miller [140]
	V170G	Rakovic [172], Seibler [170]
	I368N	Abdul [173]
*PARK7*	E64D	Burbulla [174]
*GBA*	N370S	Novosadova [161], Fernandes [175], Kim [176], Lang [177], Schöndorf [72], Straniero [178]
	L444P	Schöndorf [72], Straniero [178]
*LRRK2* + *GBA*	G2019S in LRRK2 and N370S in GBA genes	Novosadova [161]
Sporadic PD		Novosadova [161], Tolosa [154], Lang [177], Soldner [179]
